# Constitutive androstane receptor: A tumor suppressor and a biomarker for favorable prognosis of liver diseases

**DOI:** 10.1016/j.gendis.2023.101198

**Published:** 2023-12-19

**Authors:** Sarah Da Won Bae, Romario Nguyen, Lawrence Yuen, Vincent Lam, Jacob George, Liang Qiao

**Affiliations:** a Storr Liver Centre, Westmead Institute for Medical Research, University of Sydney, and Westmead Hospital, Westmead, NSW 2145, Australia; b Department of Surgery, Westmead Hospital, Westmead, NSW 2145, Australia

Constitutive androstane receptor (CAR) is a nuclear receptor that is encoded by the gene *NR1I3* (nuclear receptor subfamily 1 group I member 3) and is almost exclusively expressed in the liver.^1^ As reported in our recent publication, CAR is well established as a xenosensor for drugs and energy metabolism with newer implications in the regulation of normal liver physiology and liver regeneration.^2^ However, many controversies exist regarding the biological roles of CAR in human liver cancer^2^ and the species difference between the role of CAR in animal and human liver cancers are evident in the existing experimental and epidemiological data. Activation of CAR in animal models facilitates pro-carcinogenic pathways, eventually leading to the development of hepatocellular carcinoma (HCC). The same phenomenon is not seen following the activation of CAR in human liver cancer models. Recently, a few studies have highlighted the possible tumor-suppressive role of CAR in human cancer including liver cancer.^3^^,^^4^ Here, we aimed to unveil the clinical implications of CAR in human HCC patients.

A total of nine publicly available microarray datasets were studied to analyze the expression pattern of CAR in non-tumor and HCC tissues from patients (Supplementary Materials and Methods). CAR was significantly down-regulated in human HCC tissue in 8 out of the 9 datasets analyzed (*P* < 0.05; Fig. 1A and Table S1). Then we analyzed the expression patterns of CAR across different tumor stages and grades in GDC TCGA LIHC and TCGA, Firehose Legacy datasets (Fig. 1B–E). A pattern of decreasing CAR expression with worsening tumor stages was observed although this was significant only between stages 1 and 3 (*P* < 0.0001, GDC TCGA LIHC; *P* = 0.0393, TCGA, Firehose Legacy) (Fig. 1B–D). A similar trend was found when CAR expression was analyzed with different tumor grades (Fig. 1C, E), however, a significant difference in CAR expression was found only between grades 2 and 3 in GDC TCGA LIHC (*P* = 0.0027; Fig. 1C). Expression of CAR was also significantly down-regulated in grade 3 HCC when compared with normal tissue (*P* = 0.0009; Fig. 1F). We explored the pattern of CAR expression across different liver diseases and discovered that CAR expression decreases as liver disease progresses (Fig. 1G). CAR expression between normal and late HCC (*P* = 0.0404), chronic hepatitis and late HCC (*P* = 0.0017), dysplastic nodules and late HCC (*P* = 0.0003), and early and late HCC (*P* = 0.0498) all showed significant difference.

**Figure 1:**
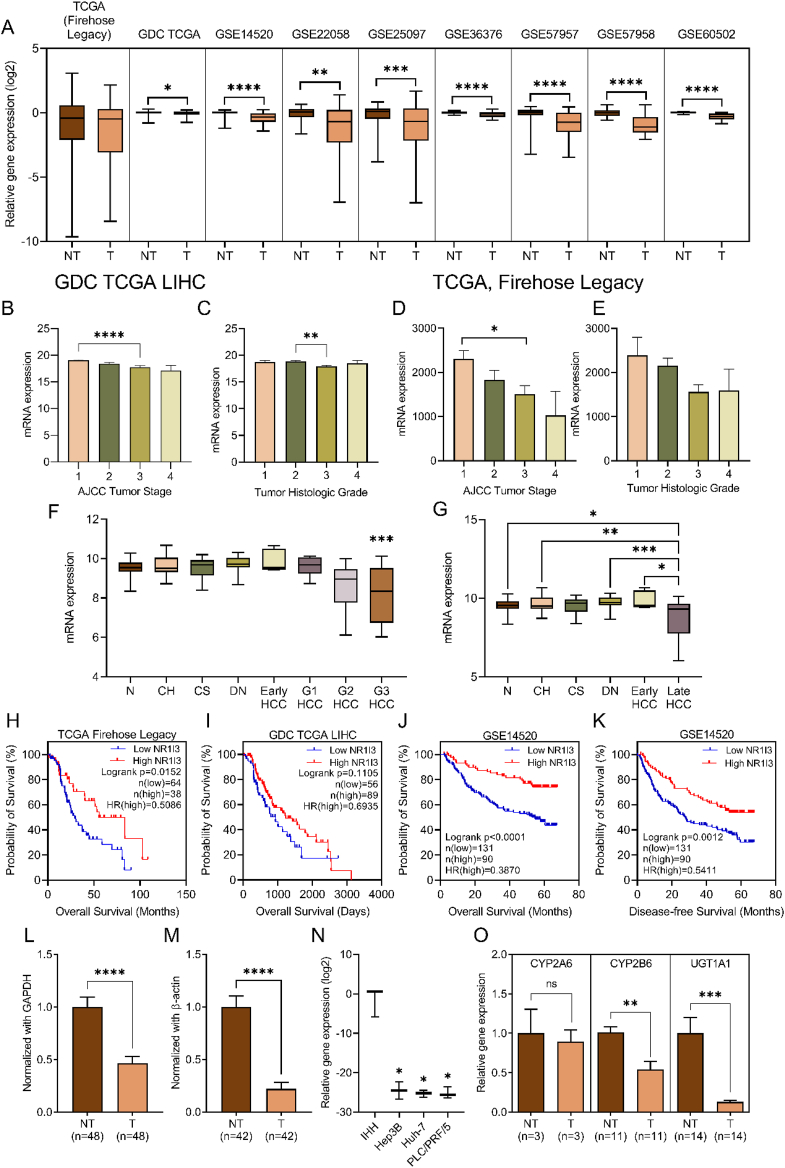
Constitutive androstane receptor (CAR) expression patterns and clinical implications in hepatocellular carcinoma (HCC) patients. **(A)** Bioinformatic analysis of CAR expression in HCC tissues and matched non-HCC tissues. CAR expression in different stages and histologic grades of HCC tissues in GDC TCGA LIHC **(B, C)** and TCGA, Firehose Legacy **(D, E)** datasets. The association of CAR expression with HCC grades and stages was further verified in the GSE89377 dataset **(F, G)**, where a significant reduction of CAR was seen only in more advanced and late-stage HCC (F) and more specifically in grades 3 and 4 HCC (G) but not in various pre-cancerous conditions. Patients with higher CAR expression (high NR1I3) showed better overall survival (OS) in TCGA Firehose Legacy **(H)**, GDC TCGA LIHC **(I)**, and GSE1450 **(J)** datasets. A better disease-free survival (DFS) was also seen in patients with higher CAR expression **(K)**. CAR expression was detected at the mRNA **(L)** and protein **(M)** levels in human HCC tissues and matched non-HCC tissues. The expression of CAR in established HCC cell lines was examined by quantitative PCR (*n* = 3), using an immortalized human hepatocyte cell line (IHH) as a control **(N)**. The expression of CAR-specific downstream targets including CYP2A6, CYP2B6, and UGT1A1 **(O)** in the same tissues as in panels A and B was examined by quantitative reverse transcription PCR. T, HCC tumors; NT, matched non-HCC tumors; N, normal liver; CH, chronic hepatitis; CS, cirrhosis; DN, dysplastic nodule; G1–3, grades 1–3 HCC; HR, hazard ratio; ns, not significant. The data were represented as mean ± standard error of the mean. ^∗^*P* < 0.05; ^∗∗^*P* < 0.01; ^∗∗∗^*P* < 0.001; ^∗∗∗∗^*P* < 0.0001.

Next, we investigated whether CAR could be a possible liver-specific prognostic marker for HCC. We analyzed survival data from three publicly available datasets (Fig. 1H–K; Supplementary Materials and Methods). In TCGA Firehose Legacy (Fig. 1H), patients with higher levels of hepatic CAR had better overall survival (*P* = 0.0152, hazard ratio/HR = 0.5086, 95% confidential interval/CI: 0.2995, 0.8638). Similarly, the same phenomenon was observed in GSE14520 (*P* < 0.0001, HR = 0.3870, 95% CI: 0.2528, 0.5924; Fig. 1J). Disease-free survival was also better in patients with high hepatic CAR expression than those with lower levels of CAR expression (*P* = 0.0012, HR = 0.5411, 95% CI: 0.3788, 0.7731; Fig. 1K).

To validate the findings from our bioinformatic analysis, we examined the expression of CAR using our patient tissue biobank samples. Our experimental findings echo the results from the bioinformatic analysis whereby CAR mRNA and protein levels were significantly lower in HCC tissues when compared with non-tumor tissues (*P* < 0.0001; Fig. 1L, M). Reduced CAR expression was also seen in established HCC cell lines relative to the immortalized human hepatocyte cell line (*P* < 0.05; Fig. 1N). Finally, we validated the functional activity of CAR in human HCC tissues by measuring the relative gene expression of several functional downstream markers of active CAR including *CYP2A6*, *CYP2B6*, and *UGT1A1*^1^^,^^2^ (Fig. 1O) and found a significant down-regulation of CYP2B6 and UGT1A1 (*P* < 0.05) gene expression in tumor tissues, indicating significantly reduced CAR activity in human HCC.

In conclusion, we have for the first time discovered the changing expression pattern of CAR across tumor status, stage, and grades along with different liver diseases. HCC patients with late-stage and advanced grades generally have reduced CAR expression and worse overall survival. The bioinformatics data demonstrating the reduced CAR expression level in HCC patients were validated in our in-house HCC tissues and cell lines. Our findings are in line with recent studies proposing that CAR is a tumor suppressor in the human brain^3^ and liver cancer.^4^ Thus, CAR is a potential novel liver-specific prognostic marker to predict the outcomes of HCC patients. Future studies are underway to elucidate the tumor-suppressive role of CAR in human HCC.

## Ethics declaration

Liver cancer tissues and matched adjacent non-cancerous liver tissues were obtained from patients undertaking liver resection in Westmead Hospital and Norwest Private Hospital. The project was approved by the Human Ethics Committee of The Westmead Institute for Medical Research [HREC/18/WMEAD/5 (5522)] and all patients provided written informed consents.

## Conflict of interests

The authors declare no conflict of interests.

## Funding

JG is supported by the Robert W. Storr Bequest to the Sydney 10.13039/100001236Medical Foundation, 10.13039/501100001774University of Sydney, Australia, and the 10.13039/501100000925National Health and Medical Research Council of Australia (10.13039/501100000925NHMRC), Australia Program and Investigator Grants (No. AAP2008983, APP1053206, APP1196492). The study was supported by project grants from Cancer Council NSW, Australia (No. APP1145008 to JG and LQ, APP1070076 to CL and LQ), 10.13039/501100001171Cancer Institute NSW, Australia grants 15/10.13039/100018716TRC/1-01 and 2021/ATRG2028, and an 10.13039/501100000925NHMRC Program Grant (No. APP1149976 to JG).
