# Supplementary material for: Constitutive androstane receptor: A tumor suppressor and a biomarker for favorable prognosis of liver diseases
Source: Genes Dis. 2023 Dec 19;11(6):101198. doi: 10.1016/j.gendis.2023.101198 (PMC11327506; doi:10.1016/j.gendis.2023.101198)
Supplement: Multimedia component 1 [file mmc1.docx]

# **Supplementary Materials and Methods**

## **Bioinformatics analysis of CAR expression and clinical implications**

RNASeq data for CAR mRNA expression in human normal liver and HCC was downloaded from the following GEO datasets: GSE14520, GSE22058, GSE25097, GSE36376, GSE57957, GSE57958, GSE60502. GEO dataset GSE89377 contained gene expression levels according to HCC stages and grades. Sample numbers and platforms for each GEO dataset used are listed in Supplementary Table 1. Microarray data and other clinical information was downloaded from GDC TCGA LIHC (n=412) (MEXPRESS: [https://mexpress.be/](https://protect-au.mimecast.com/s/K1WKCr8DLRtK98Vli7YT2e?domain=mexpress.be)) and LIHC TCGA Firehose Legacy (n=442) (cBioPortal: [https://www.cbioportal.org/](https://protect-au.mimecast.com/s/m45oCvl0PoCmX7NztXzvdB?domain=cbioportal.org)). Prior to downloading data from MEXPRESS, filters were applied to omit samples with null expression of NR1I3 and to only include cases of primary liver cancer.

Data for patient overall survival (OS) and disease-free survival (DFS) and their respective hepatic NR1I3 mRNA expression levels were downloaded from GDC TCGA (OS only), TCGA, Firehose Legacy and GSE14520. Samples were arbitrarily split into two groups: low NR1I3 expression (NR1I3^Low^) and high NR1I3, expression (NR1I3^High^) using Cutoff Finder (<https://molpathoheidelberg.shinyapps.io/CutoffFinder_v1/>). Survival rates were compared between NR1I3^Low^ and NR1I3^High^ samples using the Kaplan-Meier method and log-rank test.

## **Table S1. Platforms and sample numbers used in this study**

| GEO Accession | Platform | NT | T |
| --- | --- | --- | --- |
| GSE14520 | GPL3921 (Affymetrix HT Human Genome U133A Array) | 220 | 225 |
| GSE22058 | GPL6793 (Rosetta/Merck Human RSTA Custom Affymetrix 1.0 microarray) | 97 | 97 |
| GSE25097 | GPL10687 (Rosetta/Merck Human RSTA Affymetrix 1.0 microarray, Custom CDF) | 243 | 243 |
| GSE36376 | GPL10558 (Illumina HumanHT-12 V4.0 expression beadchip) | 193 | 240 |
| GSE57957 | GPL10558 (Illumina HumanHT-12 V4.0 expression beadchip) | 39 | 39 |
| GSE57958 | GPL8490 (Illumina Human Methylation 27 beadchip (HumanMethylation27_270596_v.1.2) | 59 | 61 |
| GSE60502 | GPL96 ([HG-U133A] Affymetrix Human Genome U133A Array) | 18 | 18 |
| GEO (gene expression omnibus), HCC (hepatocellular carcinoma) | | | |

NT: non-tumours; T: HCC tumours

**Human tissues**

Liver cancer tissues and matched adjacent non-cancerous liver tissues were obtained from patients undertaking liver resection in Westmead Hospital and Norwest Private Hospital. The project was approved by the Human Ethics Committee of The Westmead Institute for Medical Research [HREC/18/WMEAD/5 (5522)] and all patients provided written informed consents.

**Confirmation of CAR expression in human HCC samples and cell lines**

Human HCC cell lines, Hep3B (Cat. No. 86062703, Lot No. 18I014), Huh-7 (Cat. No. JCRB0403, Lot. No. 08282017), and PLC/PRF/5 (Cat. No. 85061113, Lot No. 10D004) were purchased from Cell Bank Australia. The immortalized human hepatocyte cell line IHH (in house) was used as a control. All cell lines (except Huh-7) were cultured in Dulbecco’s Modified Eagle Medium (Lonza) with 10% fetal bovine serum (FBS). Huh-7 was cultured using Dulbecco’s Modified Eagle Medium with low glucose (Sigma-Aldrich) supplemented with 10% FBS. Total RNA was extracted from cells using the FavorPrep Tissue Total RNA Kit (Favorgen Biotech Corporation) according to the manufacturer’s protocol and was used to generate cDNA using M-MLV reverse transcriptase (Promega). QuantiNova SYBR Green PCR Kit (Qiagen) was used for RT-qPCR using the recommended cycling conditions on the CFX96 Touch Real-Time PCR Detection system (Bio-Rad). Data was analyzed using the double delta Ct method.

**Table S2. Primer sequences used in the study**

| **Genes** | **Direction** | **Primer sequence** |
| --- | --- | --- |
| GAPDH | F  R | GTGGTCTCCTCTGACTTCAAC  ATTCGTTGTCATACCAGGAAATG |
| CAR | F  R | TGGCATGAGGAAAGACATGATAC  GATCAGCTCTTCTTGCTCCTTAC |
| CYP2A6 | F  R | TTTTGGTGGCCTTGCTGGT  GGAGTTGTACATCTGCTCTGTGTTCA |
| CYP2B6 | F  R | AAGCGGATTTGTCTTGGTGAA  TGGAGGATGGTGGTGAAGAAG |
